# Response of soil organic carbon and nitrogen to nitrogen deposition in a *Larix principis-rupprechtii* plantation

**DOI:** 10.1038/s41598-018-26966-5

**Published:** 2018-06-05

**Authors:** Junyong Ma, Fengfeng Kang, Xiaoqin Cheng, Hairong Han

**Affiliations:** Key laboratory of ministry of Forest Cultivation and Conservation of Ministry of Education, Beijing Forestry University, Beijing, 100083 China

## Abstract

Plant growth and ecosystem production are limited by nitrogen (N), however, the mechanisms of N limitation in terrestrial carbon (C) sequestration in soil remains unclear. To examine these mechanisms N was deposited at rates of 0, 50, 100, and 150 kg N ha^−1^ yr^−1^ for two years in a subalpine *Larix principis-rupprechtii* plantation. Soil C and N components were measured three times encompassing the entire growing season (spring, summer, and autumn) in the second year of the experiment. Results showed that N-deposition affected soil organic carbon (SOC) in the upper soil layer (0–10 cm) especially in the summer season. Dissolved organic carbon (DOC) played the key role in C loss under the high-N treatment (p < 0.01) with higher N-deposition significantly increasing both DOC and DOC/SOC in summer (p < 0.01). In the summer season when there was sufficient precipitation and higher temperatures, the average DOC across all treatments was higher than spring and autumn. The active C components contributed to SOC sequestration in low and medium N- treatment and DOC, DON dynamics in summer were responsible for the C and N pool loss under the high N-treatment.

## Introduction

Human activities have significantly increased the generation and deposition of nitrogen (N) and its active components^1,2^. Atmospheric N deposition primarily from the combustion of fossil fuels and artificial fertilizer application^3^ has increased three to five times over the last century^4,5^, exceeding N inputs from natural sources^6^. Forest ecosystems sequester nearly 30% of global CO_2_ emissions and represent one of the largest global C pools in terrestrial ecosystems. Forest ecosystems are crucial for mitigating climate change^7^ and play an important role in the global carbon (C) cycle^5,8^. Previous researches had reported that C and N cycles are intimately coupled in forest ecosystems^9,10^. A strong positive correlation of net C sequestration with N-deposition in temperate and boreal forests^11^, however, how N-deposition impacts the terrestrial C pool, one of the most important drivers of global change, needs further study^12–14^.

Nitrogen deposition may affect vegetation C pools through changing plant growth as a result of availability of soil nutrients, reducing N limitation and increasing net primary productivity of ecosystems^15,16^. Nitrogen deposition may also cause soil acidification, stimulating nitrate leaching^17^, which can increase through hydrological processes^18^ and reduce C and N pool. Additionally, N saturation could negatively affect plant growth^19,20^. Recent research also indicates that N-deposition may affect microbial activity^21,22^, litter and root biomass decomposition^23^, and soil respiration^24^. Many studies have shown that a large amount of N-deposition may hinder decomposition of soil organic matter and in turn increase soil C^25–27^.

The soil organic C pool is composed of various organic C components which have different turnover rates and chracteristics^28^. Thus, certain organic components of the C pool respond to resource availability differently^29,30^. Blair^31^ divided the C pool into an active C group and refractory C group and pointed out that the active C plays an important role in balancing soil C and nutrient flow. Biederbeck^32^ also suggested that the conversion of the C pool is mainly a result of oxidation and decomposition indicating that the active C components play a key role in C sequestration and nutrient flow. Permanganate oxidizable C (POXC) is more sensitive to changes in soil physical and chemical properties than SOC^33^ and is considered an early indicator of C dynamics^34^. Dissolved organic carbon (DOC) and dissolved organic nitrogen (DON) in soil are vital to soil C and nutrient cycling. These components are active in the physical movement and chemical transformation^35^ of C and nutrients. DOC and DON are sensitive to seasonal changes, soil properties, fertilizer addition, and land use change. DOC and DON are the intermediate link between SOC mineralization^36^ and are considered one of the most important pathways of soil carbon loss^37^ in forest ecosystems. DOC and DON content can reflect the stability of SOC^38^. Another active C component, microbial biomass carbon (MBC), is a sensitive biological index of environmental change^39^.

In order to provide a reference of C dynamics under increasing atmospheric N-deposition, an N-deposition experiment was conducted in a *Larix principis-rupprechtii* plantation in the montane secondary forest of Shanxi Province, North China. TN (total nitrogen), SOC, POXC, DOC, DON, and MBC at depths of 0–10 and 10–20 cm under four different N-deposition treatments at three different times across the growing season were analyzed. The specific objectives of this study were to determine: (1) how SOC and TN vary under different levels N-deposition; (2) how active components of soil C and N interact with each other under different N-deposition treatments; (3) and the mechanisms leading to changes in the C and N pool.

## Results

### General characteristics of the soil

**Figure 1:**
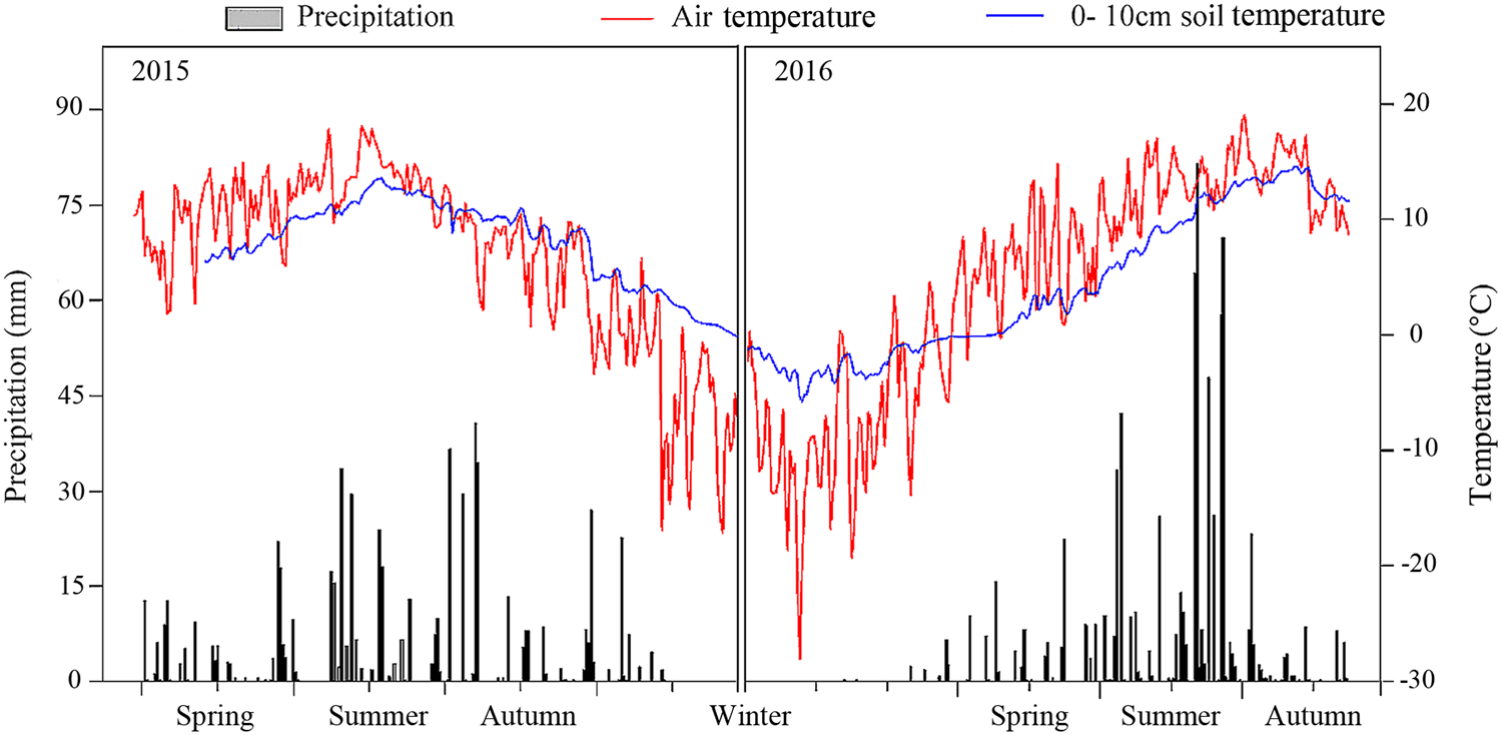
Air temperature, 10 cm soil temperature and average precipitation in the study area from spring 2015 to autumn 2016.

Stand and site characteristics were measured prior to N- addition including SOC, TN, moisture content, bulk density, pH, and soil depth (Table 1). Understory species composition was relatively simple in this *L. principis-rupprechtii* plantation, containing 28 families, 56 genera and 70 species. The dominant species included in the herb layer were *Spodiopogon sibiricu*, *Carex rigescen, Duchesnea indica, Dendranthema chanetii* and the shrub layer were *Spiraea pubescen, Lonicera japonica, Rosa xanthine* and *Lonicera serreana* (Attached Table 1). Soil temperature, air temperature, and precipitation were higher in summer compared with spring and autumn (Fig. 1).

**Table 1:** Means ± standard error (n = 3) of initial soil chemical properties (0–30 cm depth) prior to N deposition. The plots were selected randomly around the sample area before 2014 July.

Stand characteristics						Site characteristics					
Age	density	Height	Slope	Slope	Average	SOC	TN	Moisture	Bulk density	pH	Soil depth
/a	/Plant·hm^−2^	/m	aspect	gradient/°	DBH/m	/(g·kg^−1^)	/(g·kg^−1^)	content/%	/(g·cm^−3^)		/cm
34	659	2276.9 ± 2.3	SW	24.3 ± 5.1	19.61	44.9 ± 3.3	4.6 ± 0.3	26.3 ± 5.3	0.9 ± 0.1	7 ± 0.1	27 ± 4

SW: south west; DBH: diameter at breast height.

### Effects of N deposition on SOC and active C components

**Figure 2:**
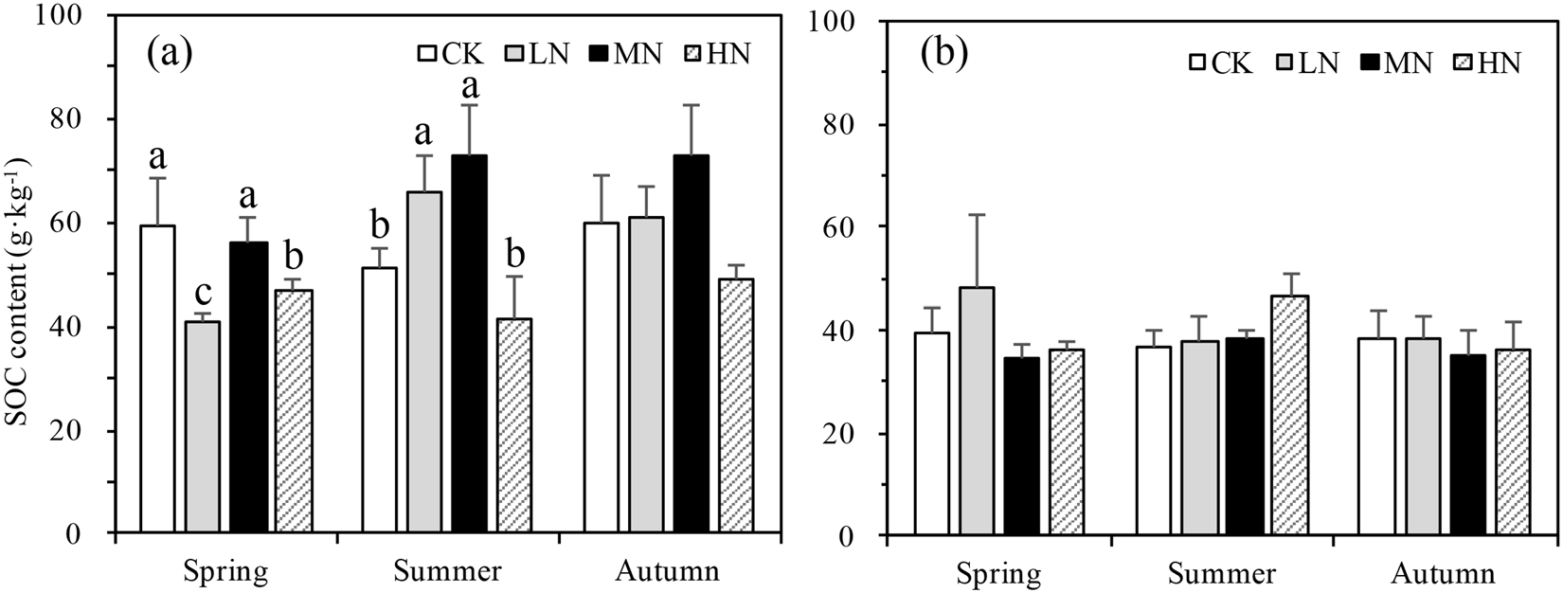
Variation in soil organic carbon (SOC) content under different N-deposition treatments in 0–10 cm (**a**) and 10–20 cm (**b**) in spring, summer, and autumn. CK: Control (0 kg N·ha^−1^ yr^−1^); LN: low-N deposition (50 kg N·ha^−1^ yr^−1^); MN: medium-N deposition (100 kg N·ha^−1^ yr^−1^); HN: high-N deposition (150 kg N·ha^−1^ yr^−1^). Each value in the plot represents the average value of three plots replicates. The error bars represent the standard error and different letters indicate significant differences among treatments (p < 0.05).

A significant three-way interaction was found in SOC between N-deposition, sampling season, and depth (p = 0.011, Table 2). The average SOC (n = 36) from the first soil layer (0–10 cm) across all three seasons and N-deposition treatments was 56.27 g·kg^−1^, 31.02% higher than the second soil layer (10–20 cm) (Fig. 2). N-deposition had a significant effect on SOC in the top soil layer in spring (p = 0.036) across the N-deposition treatments with CK (59.16 g C kg^−1^) ≥ MN (55.98 g C kg^−1^) > HN (46.18 g C kg^−1^) > LN (40.68 g C kg^−1^). A significant trend was also observed in summer (p = 0.012) however it differed by N-deposition treatment with MN (72.71 g C kg^−1^) ≥ LN (65.63 g C kg^−1^) > CK (50.97 g C kg^−1^) ≥ HN (46.39 g C kg^−1^). The trend in autumn was not significant (p = 0.081). N-deposition had no significant effect on SOC in the second soil layer in any season (Fig. 2). The average SOC from 0–20 cm crossing vegetative seasons was 47.45 g·kg^−1^ in the control plots and 48.61 g·kg^−1^ for LN, 42.60 g·kg^−1^ for HN and 51.52 g·kg^−1^ for MN. SOC increased significantly with the growing season and SOC was 9.01% higher in autumn than spring on average of the data pooled from the soil layers and N-treatments (Fig. 2).

**Table 2:** Three-way ANOVA on soil carbon and nitrogen components and soil pH in the 0–10 and 10–20 cm soil depth. n = 72 i.e.

Treatments	SOC	POXC	DOC	MBC	TN	DON	pH
Sampling seasons	0.189	0.417	<0.001	<0.001	0.332	<0.001	0.138
N- addition treatments	0.012	<0.001	<0.001	0.059	0.001	0.518	0.001
Depth	<0.001	<0.001	0.537	<0.001	<0.001	0.031	<0.001
N- addition * seasons	0.306	0.165	<0.001	0.049	<0.001	<0.001	0.192
Seasons * depth	0.027	0.412	0.198	<0.001	0.564	0.193	0.749
N- addition * depth	<0.001	0.149	0.224	0.528	0.049	0.05	0.931
N- addition * seasons * depth	0.011	0.001	0.518	<0.001	<0.001	0.097	0.011

Three sampling seasons * four N- addition levels * two soil depths * three repetitions, the values in the table are p-values.

**Figure 3:**
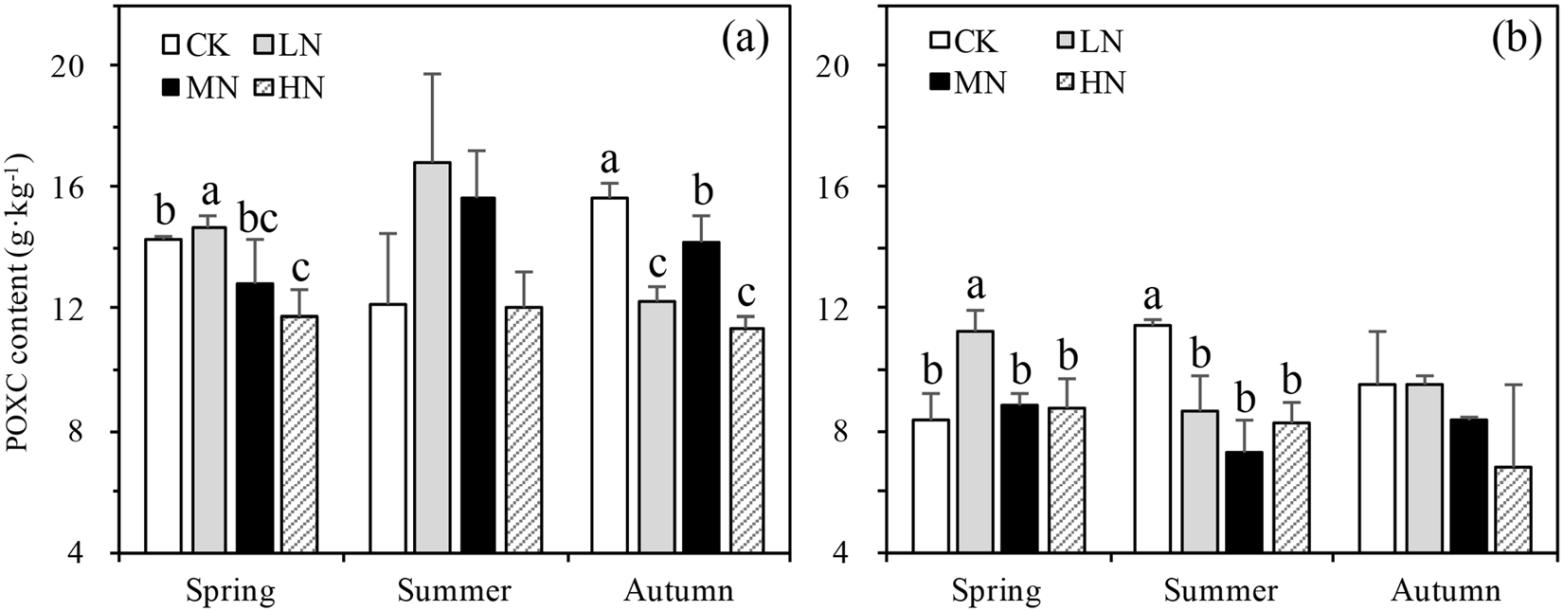
Variation in permanganate oxidizable carbon (POXC) content in different N-deposition treatments in 0–10 cm (**a**) and 10–20 cm (**b**) in spring, summer, and autumn. CK: Control (0 kg N·ha^−1^ yr^−1^); LN: low-N deposition (50 kg N·ha^−1^ yr^−1^); MN: medium-N deposition (100 kg N·ha^−1^ yr^−1^); HN: high-N deposition (150 kg N·ha^−1^ yr^−1^). Each value in the plot represents the average value of three plots replicates. The error bars represent the standard error and different letters indicate significant differences among treatments (p < 0.05).

A significant three-way interaction was found in POXC between N-deposition, sampling season, and depth (p = 0.001, Table 2). In the spring season POXC was affected significantly by N-deposition treatment in both the first (p = 0.031) and the second (p = 0.016) soil layers. POXC content was highest in the LN treatment (Fig. 3) in spring for both the first and the second soil layer. POXC decreased by 34.35% from 0–10 to 10–20 cm across all treatments and season. POXC decreased 5.1% from summer to autumn and POXC increased the most in summer (Fig. 3).

**Figure 4:**
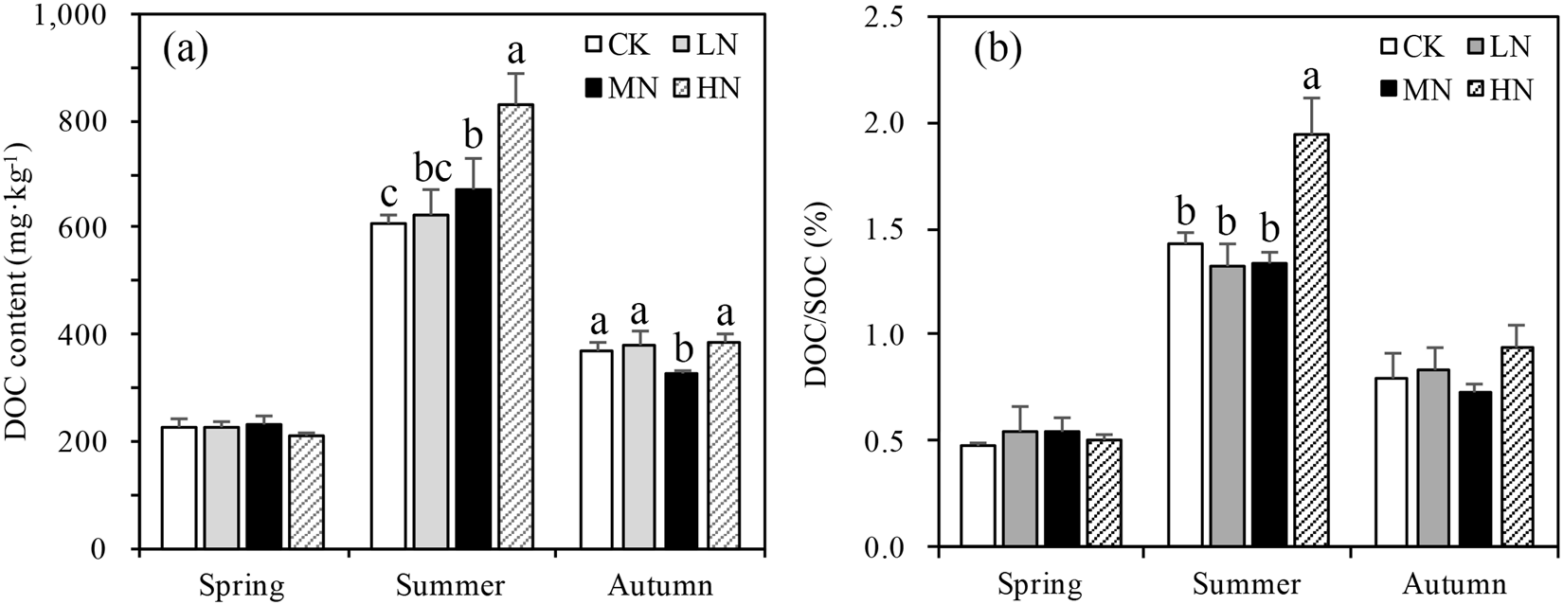
Variation in dissolved organic carbon (DOC) content (**a**) and DOC/SOC (**b**) in different N-deposition treatments in 0–20 cm in spring, summer, and autumn. CK: Control (0 kg N·ha^−1^ yr^−1^); LN: low-N deposition (50 kg N·ha^−1^ yr^−1^); MN: medium-N deposition (100 kg N·ha^−1^ yr^−1^); HN: high-N deposition (150 kg N·ha^−1^ yr^−1^). Each value in the plot represents the average value of three plots replicates. The error bars represent the standard error and different letters indicate significant differences among treatments (p < 0.05).

The DOC content showed no significant vertical change and no interaction was found among the three variable factors except N-deposition with seasons (p < 0.001) (Fig. 4a and Table 2). When analyzed separately, N-deposition affected DOC significantly in summer (p = 0.007) and autumn (p = 0.042) (Fig. 4a). In summer with increasing N-deposition DOC increased reaching a maximum under high-N deposition. In spring DOC was not affected by N-deposition treatments. DOC/SOC was highest in high N-deposition compared to the other treatments in the summer (p < 0.001) (Fig. 4b).

**Figure 5:**
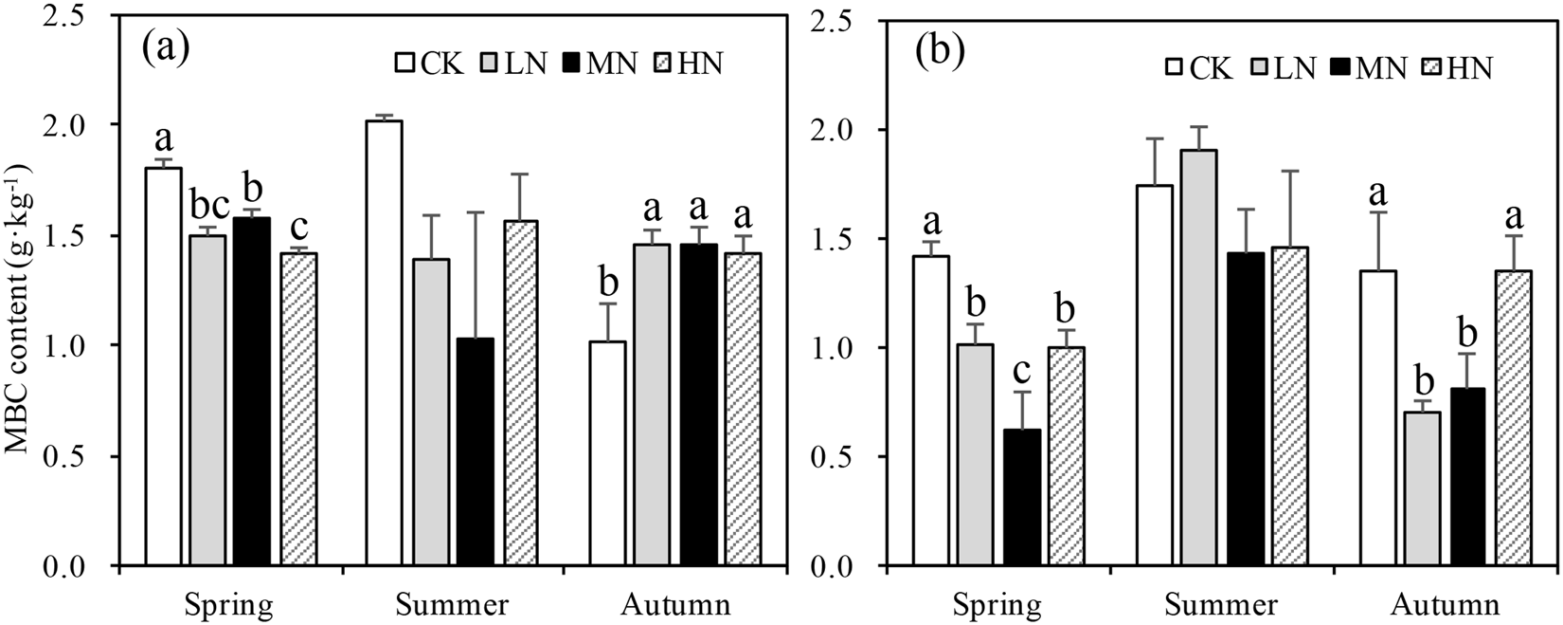
Variation of microbe biomass carbon (MBC) content in different N-deposition treatments in 0–10 cm (**a**) and 10–20 cm (**b**) in spring, summer, and autumn. CK: Control (0 kg N·ha^−1^ yr^−1^); LN: low-N deposition (50 kg N·ha^−1^ yr^−1^); MN: medium-N deposition (100 kg N·ha^−1^ yr^−1^); HN: high-N deposition (150 kg N·ha^−1^ yr^−1^). Each value in the plot represents the average value of three plots replicates. The error bars represent the standard error and different letters indicate significant differences among treatments (p < 0.05).

N-addition coupled with soil depth and sampling seasons significantly affected MBC (p < 0.001, Table 2). MBC content in summer was significantly higher than in other seasons (Fig. 5 and Table 2).

### Effects of N deposition on TN and nitrogen components

**Figure 6:**
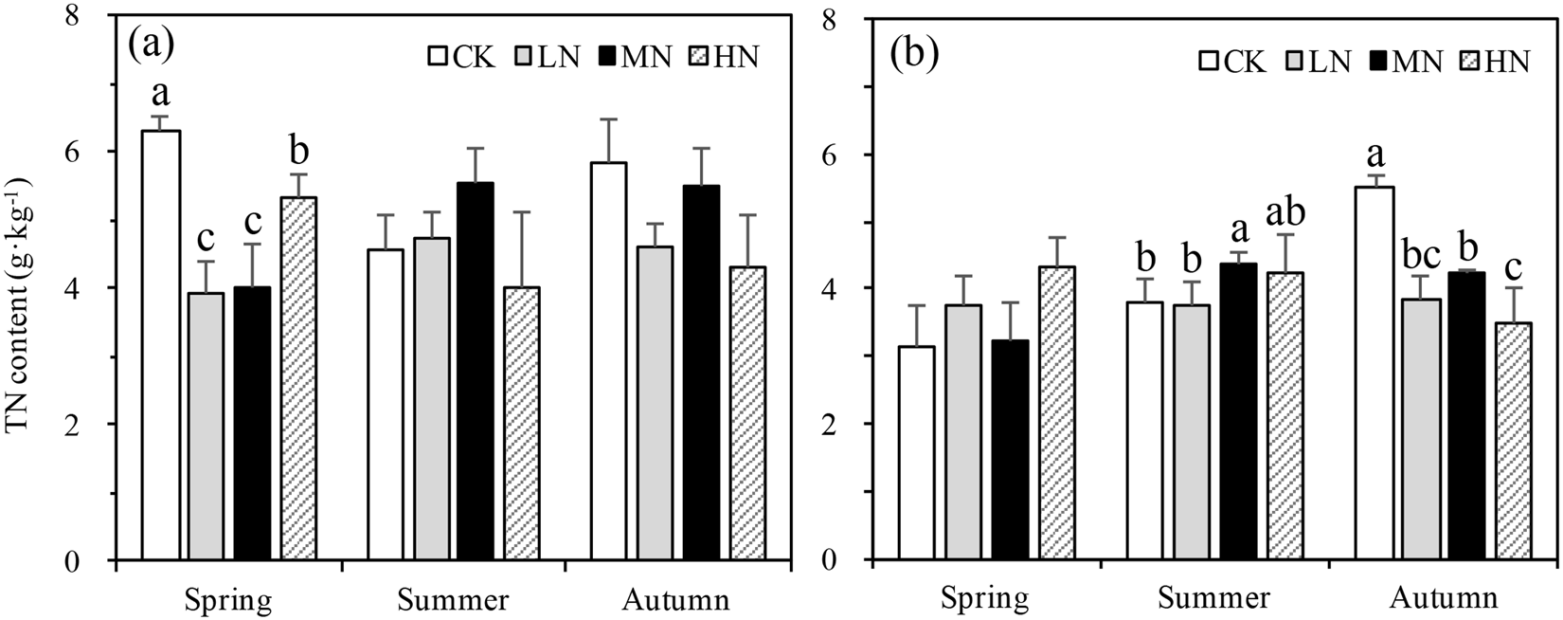
Variation of total nitrogen (TN) content in different N-deposition treatments in 0–10 cm (**a**) and 10–20 cm (**b**) across vegetative seasons. CK: Control (0 kg N·ha^−1^ yr^−1^); LN: low-N deposition (50 kg N·ha^−1^ yr^−1^); MN: medium-N deposition (100 kg N·ha^−1^ yr^−1^); HN: high-N deposition (150 kg N·ha^−1^ yr^−1^). Each value in the plot represents the average value of three plots replicates. The error bars represent the standard error and different letters indicate significant differences among treatments (p < 0.05).

A significant three-way interaction was found in TN between N-deposition, sampling season, and depth (p < 0.001, Table 2). N-deposition had a significant impact on TN in both summer (p = 0.006) and autumn (p = 0.001) in the second soil layer (Fig. 6b). N-deposition affected TN in the first soil layer only in spring (p = 0.002, Fig. 6a). The pooled TN data for all three seasons showed decreases of 18.59%, 16.12%, and 14.15%, in the low, medium, and high N-deposition treatments compared with control plots (Fig. 6). TN decreased significantly (p < 0.001) by 32.7% from 0–10 to 10–20 cm in all the three seasons. TN increased significantly with the growing season. In autumn TN increased 7.2% compared to spring (Fig. 6).

**Figure 7:**
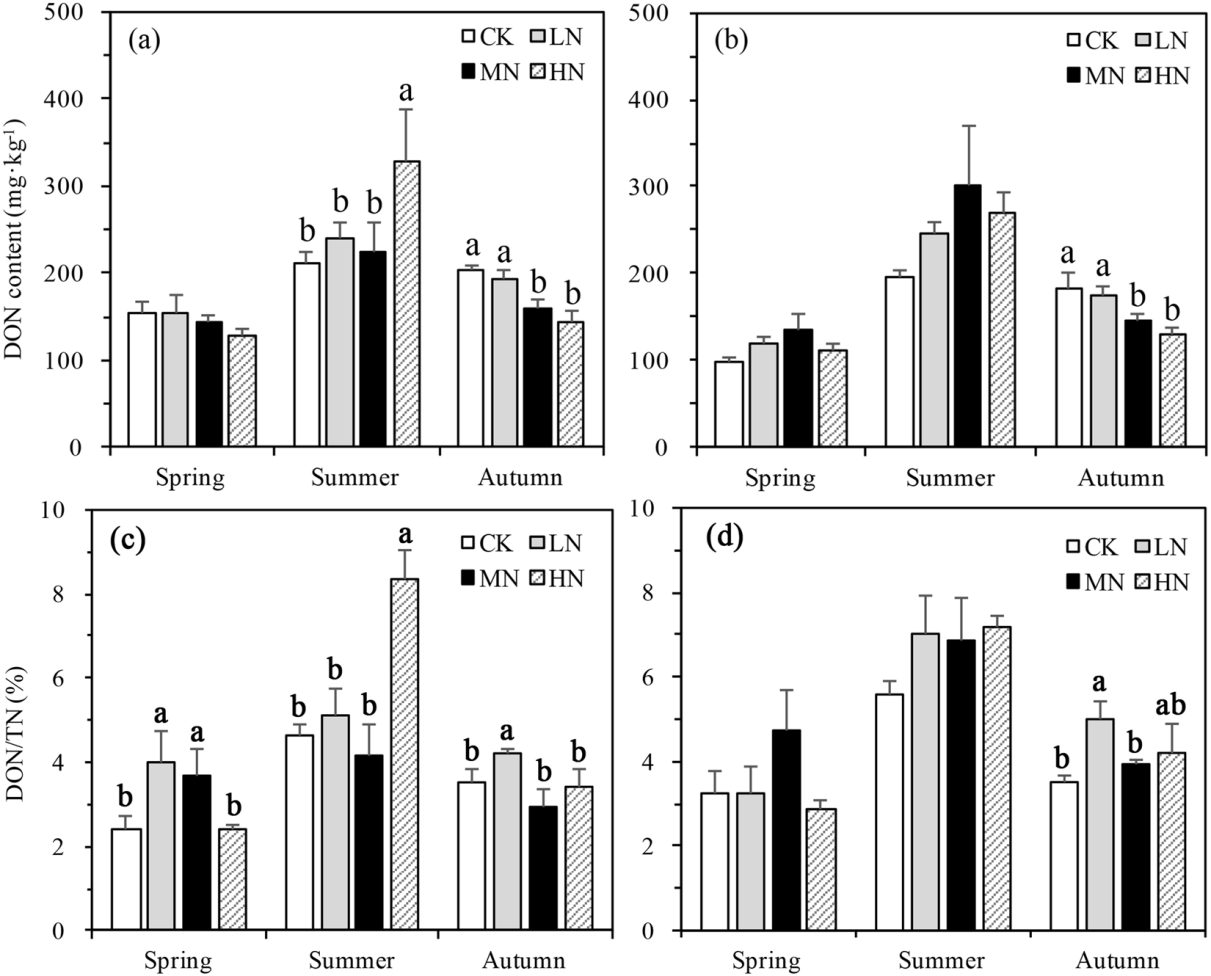
Variation of dissolved organic nitrogen (DON) and DON/TN content in different N-deposition treatments in 0–10 cm (**a** for DOC, **c** for DON/TN) and 10–20 cm (**b** for DOC, **d** for DON/TN) across vegetative seasons. CK: Control (0 kg N·ha^−1^ yr^−1^); LN: low-N deposition (50 kg N·ha^−1^ yr^−1^); MN: medium-N deposition (100 kg N·ha^−1^ yr^−1^); HN: high-N deposition (150 kg N·ha^−1^ yr^−1^). Each value in the plot represents the average value of three plots replicates. The error bars represent the standard error and different letters indicate significant differences among treatments (p < 0.05).

No significant three-way interaction was found in DON between N-deposition, sampling season, and depth (p = 0.097, Table 2). DON changed significantly from 0–10 cm to 10–20 cm (p = 0.031, Table 2). In autumn, DON decreased significantly with N-deposition in both the first (p = 0.001) and the second (p = 0.004) soil layer. In the first layer DON was significantly higher in the summer (p = 0.046) under HN (328 mg N kg^−1^) deposition than MN (225 mg N kg^−1^), LN (240 mg N kg^−1^) or CK (211 mg N kg^−1^). DON content in summer (251 mg N kg^−1^, all the data pooled from summer) was higher than in spring (130 mg N kg^−1^) and autumn (167 mg N kg^−1^). DON was 31.9% higher in the treatment groups (low, medium and high N deposition) than no N-deposition in summer on an average across all three treatments (Fig. 7a,b). The N-deposition treatments affected DON/TN significantly in the first soil layer across the seasons, in summer DON/TN in the HN treatments was extremely higher the other treatments (Fig. 7c). However, DON/TN in the second soil layer was affected by N-deposition treatments significantly only in autumn (Fig. 7d).

### Relationships between soil C and N under N deposition

The C/N ratio (SOC/TN) in the second soil layer in summer (p = 0.013) of HN plots was lower than other N-deposition treatments (Table 3); in autumn (p = 0.001), C/N was lowest in CK plots. Similar but more variable trends were found for POXC/TN ratio in spring and summer. POXC/TN in the first soil layer in spring (p = 0.005), second soil layer in summer (p = 0.002) and autumn (p = 0.096), increased when N-deposition increased from CK to low N-deposition then decreased from low to high N-deposition (Table 3).

**Table 3:** Means ± standard error (n = 3) of soil chemical properties (0–10 cm and 10–20 cm depth) under N-deposition across the growing season.

0–10 cm	C/N			POXC/TN			10–20 cm	C/N			POXC/TN		
	Spring	Summer	Autumn	Spring	Summer	Autumn		Spring	Summer	Autumn	Spring	Summer	Autumn
CK	9.7 ± 1.8a	11.3 ± 1.4a	10.3 ± 1.6a	2.3 ± 0.1c	2.6 ± 0.2a	2.7 ± 0.3a	CK	12.8 ± 1.5a	12.1 ± 0.6bc	7.3 ± 0.6c	2.7 ± 0.4a	3.8 ± 0.6a	1.8 ± 0.2a
LN	10.6 ± 1.9a	13.8 ± 0.5a	13.1 ± 0.5a	3.8 ± 0.5a	3.5 ± 0.4a	2.7 ± 0.2a	LN	12.6 ± 2.3a	13.5 ± 0.6a	12.6 ± 0.4ab	3.1 ± 0.4a	3.1 ± 0.2a	3.2 ± 0.3a
MN	14.3 ± 2.7a	13.1 ± 0.6a	13.1 ± 0.6a	3.3 ± 0.5b	2.8 ± 0.1a	2.6 ± 0.1a	MN	15.9 ± 6.3a	12 ± 0.2b	11.8 ± 0.1b	3.9 ± 1.1a	2.3 ± 0.2b	2.9 ± 0.4a
HN	8.8 ± 0.4a	10.7 ± 2.3a	11.7 ± 2.0a	2.2 ± 0.2c	3.2 ± 0.9a	2.7 ± 0.5a	HN	11.2 ± 2.5a	10.5 ± 1.0c	13.5 ± 0.6a	2.7 ± 0.6a	1.9 ± 0.2b	2.4 ± 0.8a
p	0.063	0.154	0.19	0.005	0.384	0.962	p	0.631	0.013	0.001	0.318	0.002	0.096

P-values are from different N-deposition treatments of the same sampling season. Significant differences (p < 0.05) among treatments are indicated by different letters.

**Figure 8:**
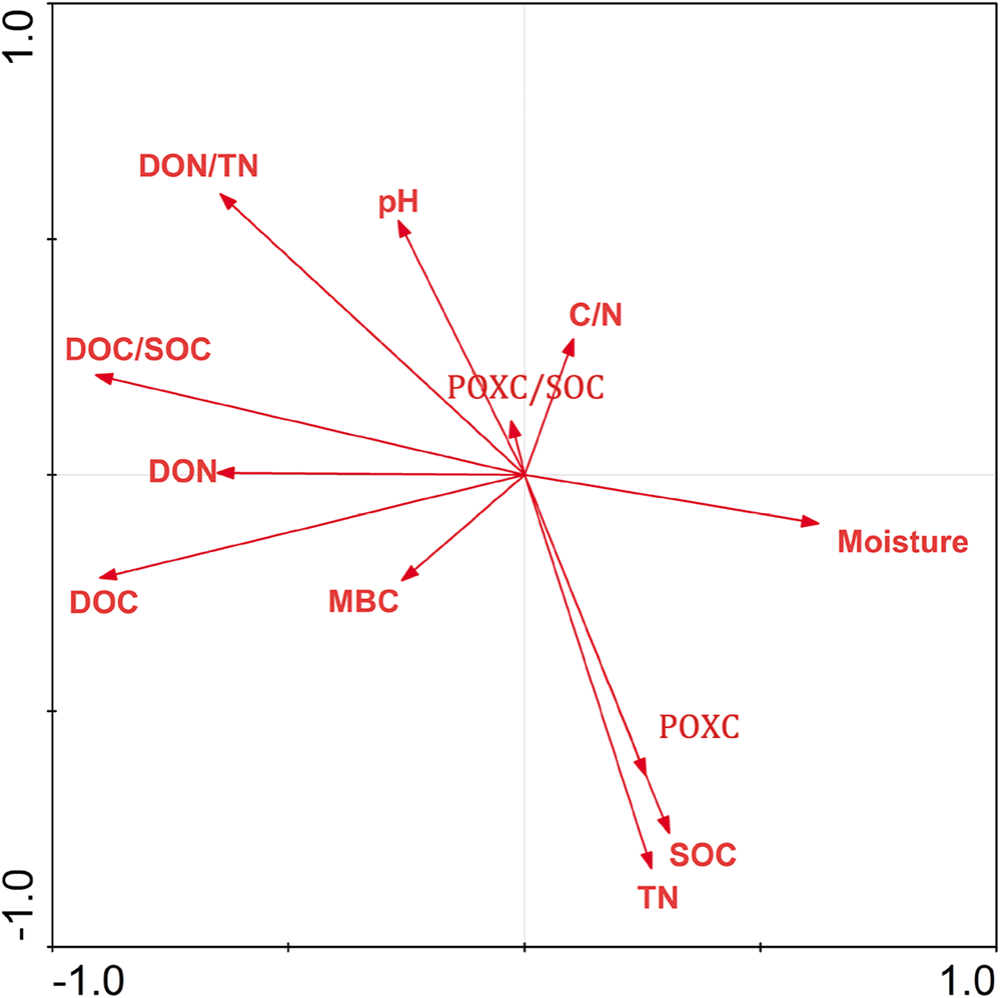
Canonical correspondence analysis (CCA) for the relationship for all the different soil properties across N-deposition treatments, seasons and soil depths. n = 72, i.e. four N-deposition treatments × three seasons × three repeats × two soil depths.

Across all N-deposition treatments, soil depth, and season a strong positive correlation was found between SOC and TN (R = 0.675, n = 72, p < 0.001) and SOC and POXC (R = 0.706, n = 72, p < 0.001) and strong negative correlations were found between pH and SOC, TN, and POXC. DOC was correlated positively with DON (R = 0.858, p < 0.001) and C/N had a negative correlation with TN (R = −0.485, p < 0.001). A significant negative correlation was found between pH and SOC (R = −0.629, n = 72, p < 0.001), TN (R = −0.653, p < 0.001), and POXC (R = −0.676, p < 0.001). DOC/SOC was significantly negatively correlated to SOC (Fig. 8).

## Discussion

The specific objectives of this study project were to determine how carbon (C) and nitrogen (N) pools vary with nitrogen deposition in a *Larix principis-rupprechtii* plantation, and how variation in each soil organic component may drive patterns in total C and N pool. Overall, SOC responded to light and medium N-deposition especially in the summer season in the first soil layer (0–10 cm), and DOC, DON dynamics in summer were responsible for the C and N pool loss under the high N-treatment.

Simulate N-deposition in agricultural ecosystems could increase SOC^40,41^. An increase in N-deposition could improve primary productivity which is the main source of soil C and N, more primary productivity may result in more carbon sequestration in forest ecosystems^11,12^, and over abundant N can create saturated conditions and result in a suppression of forest C sequestration^14,15^.

In this study, SOC was greatest in medium N-deposition in summer and autumn, but results were only significantly different from control plots in summer. Summer initiated the C cycle under different N-deposition treatments and the organic C components were the key factors driving C pool dynamics. In the summer season temperatures (both soil and air temperatures) and precipitation gradually increased (Fig. 1). Higher temperatures can enhance microbial growth^42^, which can then be further facilitated by higher concentrations of DOC providing more nutrients for microbial growth^43^. The N-deposition affected in SOC mainly in the first soli layer (Fig. 2).

The more SOC in the first soil layer could be due to the more sediment from the litter or root sourced from the upper ground, and N-deposition affected SOC mainly in the first soil layer could be due to the N-deposition affected C pool gradually and it take time to accumulate. In the medium N-deposition plots, SOC generally accumulated with the growing seasons (Fig. 2), suggested more C was sequenced in the MN treatment plots.

Soil carbon pools are mainly influenced by the balance between input from plants and loss from leaching of active carbon component like DOC^44,45^ or the release of CO_2_^46^. Previous research had found that N fertilizer application could increase DOC over short time scales^47^ but after SOC achieves stability, N fertilizer cannot further improve soil DOC content^48,49^. It is generally believed that DOC increases with seasonal increases in soil temperature and enhanced microbial activity^48^. In this study, twice as much DOC was measured in the summer than the other two seasons (Fig. 4) and both the DOC and DOC/SOC was influenced by N-deposition treatments. In the summer the DOC increased significantly with increasing N-deposition.

Water condition and temperatures which are directly influenced by seasons, have close relationships with the DOC content. Soil moisture content affects the rhizosphere and soil microbial activity, which affects soil DOC content^49^. Many studies showed that flood water can also increase the content of DOC^49,50^. SOC loss through DOC leaching in the rainy season may have caused SOC decreases in high N-deposition plots, which had both the highest DOC and DOC/SOC in the summer season (Fig. 4). In spring when the temperatures were relatively lower and the soil experienced freeze-thaw patterns (Fig. 1), and in autumn with more litter input, N-deposition had a more complex effect on SOC, DOC and DOC/SOC than the summer season (Figs 2 and 4).

Permanganate oxidizable C affects the C pool with a relatively short turnover time as it is easily oxidized and has high chemical activity^48,51^. Three-way ANOVAs indicated that N-deposition, soil depth, and sampling season all interacted presenting complex dynamics in POXC content. In the vegetative seasons significant positive correlations between POXC, SOC and TN were found indicating that POXC is closely related to the C and N pool and may affect C and N storage in soil. POXC content under low N-deposition increased most in spring and summer but when N-deposition exceeded 100 kg N∙ha^−1^∙yr^−1^, a decrease in POXC occurred (Fig. 3), but it was only statistically significant in spring.

As DOC component is an important part of the POXC (DOC/POXC was 3.8% on average), the POXC components decreased through the loss of DOC in medium and high N-deposition may be one reason for the decline of POXC in these N-deposition treatments. The decrease of POXC in autumn was most likely caused by POXC leaching through DOC, thus partially explaining the decline of POXC.

As N was added, in some treatments and seasons, TN content decreased which may be related to the addition of fertilizer- NH_4_NO_3_^52^. A proper N-deposition has been shown to accelerate nitration, increasing the loss of NO_3_-N and in turn TN concentrations^52^. Additionally, NH_4_NO_3_ acidified soil may cause ionic imbalance^17^, stimulating nitrate leaching, increasing the N pool loss through hydrological processes^18^.

The highest DON and DON/TN in the summer season in HN in first soil layer suggests N-deposition changes the characteristics of the N pool, making more DON under this treatment. Higher DON/TN in HN indicated more DON in one-unit N pool, increasing the possibility for N to loss through water leaching. High precipitation is reported to be an important factor affecting the N pool^53^ and resulting continuous N losses (via higher DON) might explain the reduction in TN in autumn.

Long-term N deposition in a hardwood forest in northern America concluded that with increasing nitrogen input significantly increasing the microbial biomass and microbial activity^54^. A recent meta-analysis also suggested C/N to be a dominant factor regulating N effects on microbial decomposition of litter and soil organic matter^53^. Here both the C/N and MBC was measured crossing plant growing seasons under N-deposition treatments. The soil C/N ratio was lowest in control plots, however only significantly in autumn. Under CK and high N-deposition C/N was low and MBC content was relatively high. In this study, a negative relationship was found between these two biological indicators (Fig. 8). Lower C/N suggests more microbial activity and higher N mineralization rates^55^. C/N ratios in forest soils tend to decline with N deposition^56^. Imbalances in C and N accumulation between N-deposition treatments and control plots may be one reason for the lower C/N ratios.

A link between MBC and POXC/SOC was also found under the different treatments with low POXC/SOC (Table 3) corresponding to less MBC. Both MBC and POXC/SOC were not significantly affected by N-deposition when the whole growing season was taken into account, indicating POXC is a sensitive indicator for C cycling^57^ and was responsible for variations in C/N.

## Conclusion

Our results show that N-deposition treatments increased C pools in the low and medium N-deposition through their active components, and the C pool dynamics primarily occurs in summer when there is abundant precipitation and warmer temperatures. The results also suggest that DOC leads to the loss of both SOC and POXC in summer which caused more C loss in the higher N-deposition treatments. Significantly higher DOC in summer under high N-treatment in the upper soil layer (0–10 cm) suggests a change in the solubility of SOC contributing to a reduction of the C pool. Lower POXC content in the higher N-deposition treatment after two years of treatment responses to the saturation effect of SOC partly. N-deposition increased DON in summer and high DON/TN during the plant growing season contributed to a loss of TN. This research suggests that N-deposition changes the C and N pool through changing the content of crucial active components of DOC and DON. Overall results suggest that low and medium N-deposition (50 and 100 kg N∙ha^−1^∙yr^−1^) in *L. principis-rupprechtii* plantations are best for forest management practices by offering more C retention.

## Materials and Methods

### Study area and experimental design

The study area located in Taiyue Mountain forest farm in Shanxi Province, Northern China (111°59′E–112°05′E, 36°40N–36°47′N). The subalpine study area is 1700–2450 m above the sea level with an average elevation of 2312.8 m. The study plot soil type is Haplic luvisols according to the FAO soil texture classification with a thickness of 20–30 cm. It is an artificial forest dominated by *Larix principis-rupprechtii* that was planted in the 1980s and has been protected since. This region has a continental monsoon climate with humid and rainy summers and cold and snowy winters. Mean annual air temperature is 8.7 °C with an average minimum temperature of −10.4 °C in January and an average maximum of 17.4 °C in July. The frost-free period lasts, on average, for 125 days, with the earliest frost generally in October and latest frost generally in April. Average annual rainfall in this region ranges between 600 and 650 mm, with precipitation occurring mainly from July to September. The dominant overstory vegetation in all stands is *L. principis-rupprechtii*. The shrub layer mainly includes *Spiraea pubescens*, *Lonicera japonica* and *Rosa xanthina*. Herbs mainly include *Spodiopogon sibiricus*, *Carex rigescens* and *Duchesnea indica*, and *Dendranthema chanetii*.

An undisturbed *Larix principis-rupprechtii* area was selected in 2014 for N-deposition. Basic soil information was tested and no obvious differences within the study area were detected (Table 1). The N-deposition experiment was initiated after basic soil information was surveyed, following a widely used method for simulating N-deposition^20^. Four N-deposition treatments were established, including control-CK plots (no N), low-N plots (LN, 50 kg N hm^−2^ yr^−1^), medium -N (MN, 100 kg N hm^−2^ yr^−1^), and high-N (HN, 150 kg N hm^−2^ yr^−1^), with three replicate plots for each treatments. Twelve 5 × 5 m plots were established. All plots and treatments were randomly laid out. During each application, the fertilizer NH_4_NO_3_ (0 g, 30 g, 60 g and 90 g NH_4_NO_3_) was weighed and dissolved in water to the desired concentration and sprayed evenly on the forest floor with a sprayer at the end of every month in the vegetation growing season from April to October.

### Sampling and chemical analyses

Mineral soil samples at 0–10 cm and 10–20 cm depths were collected in spring, summer and autumn of 2015. Three sampling points from the same plot in the treatments were randomly selected. Soils from same depth were mixed to form a composite sample for lab analyses. Visible stones and roots were removed and samples were sieved through a 2-mm mesh. After sifting, each composite soil sample was divided into two subsamples, one subsample was stored in a 4 °C incubator for later DOC and MBC content determination (analysed within 72 hours), the second subsample was air-dried and passed through a 0.25 mm sieve before analysis for SOC, total N, and POXC content.

SOC and TN were analyzed by dry combustion using an elemental analyzer (Thermo Scientific FLASH 2000 CHNS/O, USA). POXC was determined using wet oxidization with 333 mmol^−1^ KMnO_4_^31,32^. MBC was determined by HCl_4_-fumigation, K_2_SO_4_ extraction method, carbon content of non-fumigated soil samples was considered as dissolved organic carbon and nitrogen (DOC, DON)^58^. Fumigated and non-fumigated soils (10.0 ± 0.5 g fresh sample) were extracted with 40 ml 0.5 mol∙L^−1^ K_2_SO_4_ (soil: extractant = 1:4) and shaken for 30 min on a reciprocal shaker at 300 r/min, then the extraction liquid was analyzed by TOC analyzer (German Jena, Multi N/C 3000). Soil pH was estimated on a 1:2.5 soil- water mixture. Gravimetric soil water content was calculated from mass loss after drying for 24 h at 105 °C separately for all the soil layers.

MBC was calculated as:

1 $${\rm{MBC}}={\rm{EC}}/{{\rm{k}}}_{{\rm{EC}}}$$

In (1) E_C_ = (organic C extracted from fumigated soils) - (organic C extracted from non-fumigated soils) and k_EC_ = 0.45^59^.

MBN was calculated as:

2 $${\rm{MBN}}={\rm{EN}}/{{\rm{k}}}_{{\rm{EN}}}$$

In (2) EN = (total N extracted from fumigated soils) - (total N extracted from non-fumigated soils) and k_EN_ = 0.54^60^.

Significant differences were determined by three-way analysis of variance (ANOVA) and the post hoc Tukey-HSD test using the statistical package, IBM SPSS 20.0. Results were expressed as mean ± SD (n = 3). The one-way analysis of variance (ANOVA) were used to compare ratios of the soil C and N components. The statistical significance for all tests was set at p < 0.05. We used Canonical correspondence analysis (CCA) to examine the interrelationships between different soil properties, following the procedures in CANOCO software for Windows 4.5 (Biometris-Plant Research International, Wageningen, Netherlands), based on the data acrossN-deposition treatments, seasons and soil depths. n = 72, i.e. four N-deposition treatments × three seasons × three repeats × two soil depths.
